# Evaluation of the Functional Treatment of Patients With Skeletal Class II Malocclusion Using Low-Level Laser Therapy-Assisted Twin-Block Appliance: A Three-Arm Randomized Controlled Trial

**DOI:** 10.7759/cureus.23449

**Published:** 2022-03-24

**Authors:** Abdulaziz Abdulhadi, Ahmad S Burhan, Mohammad Y Hajeer, Omar Hamadah, Ghiath Mahmoud, Fehmieh R Nawaya, Mohammad Osama Namera

**Affiliations:** 1 Department of Orthodontics, Faculty of Dentistry, Damascus University, Damascus, SYR; 2 Department of Oral Medicine, Faculty of Dentistry, Damascus University, Damascus, SYR; 3 Department of Pediatric Dentistry, Faculty of Dentistry, Syrian Private University, Damascus, SYR

**Keywords:** mandibular length, duration of treatment, temporomandibular joint, mandibular retrognathism, tmj morphology, acceleration of bone formation, twin block, low-level laser therapy, class ii malocclusion, functional treatment

## Abstract

Background

Different techniques have been used to reduce functional treatment time including low-level laser therapy (LLLT), and the majority of studies have been conducted on animals. Therefore, the aim of the current study was to evaluate the effects of LLLT on improving orthodontic functional treatment using the Twin-Block (TB) appliance.

Materials and methods

This study was a three-arm, parallel-group randomized controlled trial. Patients were selected using the following inclusion criteria: skeletal Class II Division 1 malocclusion resulting from mandibular retrognathia (angle between the anterior cranial base and the NB plane (i.e., SNB angle): 73°-78°), the sagittal skeletal discrepancy angle (ANB angle) between 4° and 9°, and overjet between 5 and 9 mm. Forty-eight patients were randomly allocated into three equal groups. In the LLLT-TB group, the low-level laser device was used with a wavelength of 808 nm and power of 250 mW in addition to functional treatment with a Twin-Block appliance. The laser was applied on the skin at the bilateral temporomandibular joint (TMJ) regions, at five points, each point received 5 J of the laser for 20 seconds. The laser course was twice a week in the first month, every two weeks in the second month, and every three weeks up to the end of the treatment. The second group (the TB group) received functional treatment with a Twin-Block appliance, while patients in the third group (the untreated control group (UCG)) were observed for nine months without any intervention.

Results

There were statistically significant differences in treatment periods between the LLLT-TB group and the TB group (129 days and 235 days, respectively, P-value<0.001). The change in the effective mandibular length (Co-Gn) was the highest in the LLLT-TB group compared with the TB and the UCG groups (4.41 mm, 3.66 mm, and 1.07 mm, respectively; P-value<0.001).

Conclusions

The application of low-level laser therapy on the condylar regions accelerated the functional treatment in skeletal Class II malocclusion patients by approximately 45% and increased the bone growth and mandibular length. The improvement in the SNB angle was similar in both interventional groups. Irradiation of low-level laser stimulated bone growth at the condyles and did not cause anterior movement of the temporomandibular joint following functional orthopedic correction.

## Introduction

Class II malocclusion is considered the most prevalent orthodontic malocclusion throughout the orthodontic practice. About 15%-20% of the world's population suffers from this problem [1]. Orthodontists have used various types of functional appliances and have shown different dentoalveolar results with various degrees of effectiveness [2]. Several techniques, such as low-level laser, ultrasound stimulation, anabolic steroids, growth hormone, and cyclosporine, have been suggested to reduce functional treatment time and stimulate the condylar cartilage and bone [3]. In recent years, the treatment with low-level laser therapy (LLLT) has gained popularity in different dental fields. It is defined as a treatment that avoids heating living tissues above the 36.5°C threshold [4], making use of the biostimulation characteristics of laser on cells because of its good ability to penetrate living tissues leading to bioreactions within the aimed cells [5].

Many studies have evaluated the effects of LLLT on the growth of the mandible and condyle in animals [6,7]. These studies have shown promising results about laser’s ability to stimulate cell proliferation in the condylar region, which affects mandibular growth [6,7]. Seifi et al. reported that particular laser irradiation can stimulate condylar growth and subsequently cause mandibular advancement in rats [7]. Abtahi et al. histologically evaluated the temporomandibular joint (TMJ) in rabbits after functional treatment assisted with LLLT and revealed that irradiation of laser during mandibular advancement increased bone formation in the condylar region, while an increase neither in the cartilage thickness nor in the fibrous tissues was observed [6]. Likewise, Saafan et al. histologically assessed the application of LLLT in rabbits and found that it enhanced mandibular growth by condylar endochondral bone growth, while it did not increase the fibrous tissue [8]. Okşayan et al. studied the stimulation and acceleration of mandibular growth with LLLT on condyle with or without functional appliances in rats [9]. They concluded that functional appliances supplemented with LLLT stimulated condylar growth and increased mandibular advancement without any untoward side effects on the TMJ region [9].

Despite the promising results obtained from animal studies, there is no clinical trial evaluating the effect of functional treatment conducted on skeletal Class II patients in conjunction with LLLT. Therefore, this trial was accomplished to assess the effects of LLLT on accelerating functional treatment and stimulating bone growth in these dentofacial deformities. The null hypothesis tested that there were no significant differences in bone growth of the mandible among those patients who were treated only with the Twin-Block appliance, those patients who were treated with the Twin-Block appliance supplemented with irradiation of the condylar region with LLLT, and those patients who were observed for nine months without treatment.

## Materials and methods

Study design and settings

The current study was designed as a three-arm, parallel-group randomized controlled trial with a 1:1:1 allocation ratio. This study was conducted at the Departments of Orthodontics and Oral Medicine, Faculty of Dentistry, Damascus University, Damascus, Syria. It was ethically approved by the Local Research Ethics Committee of Faculty of Dentistry, Damascus University (UDDS-485-01512018/SRC-2139). The trial was registered at ClinicalTrials.gov (NCT03239912) before the enrollment of the first patient in the study.

Sample size calculation

G*Power software version 3.1.3 (University Kiel, Germany) was used to calculate the sample size using the following criteria: the effect size for the angle between the anterior cranial base and the NB plane (SNB) angle change between T2 and T1 according to Giuntini et al. [10] was 0.581, the power of 90%, an alpha level of 0.05, and one-way ANOVA as a statistical test. The required sample size was 14 patients in each group, and it was increased to 16 patients to compensate for any potential dropouts.

Participants and eligibility criteria

**Figure 1:**
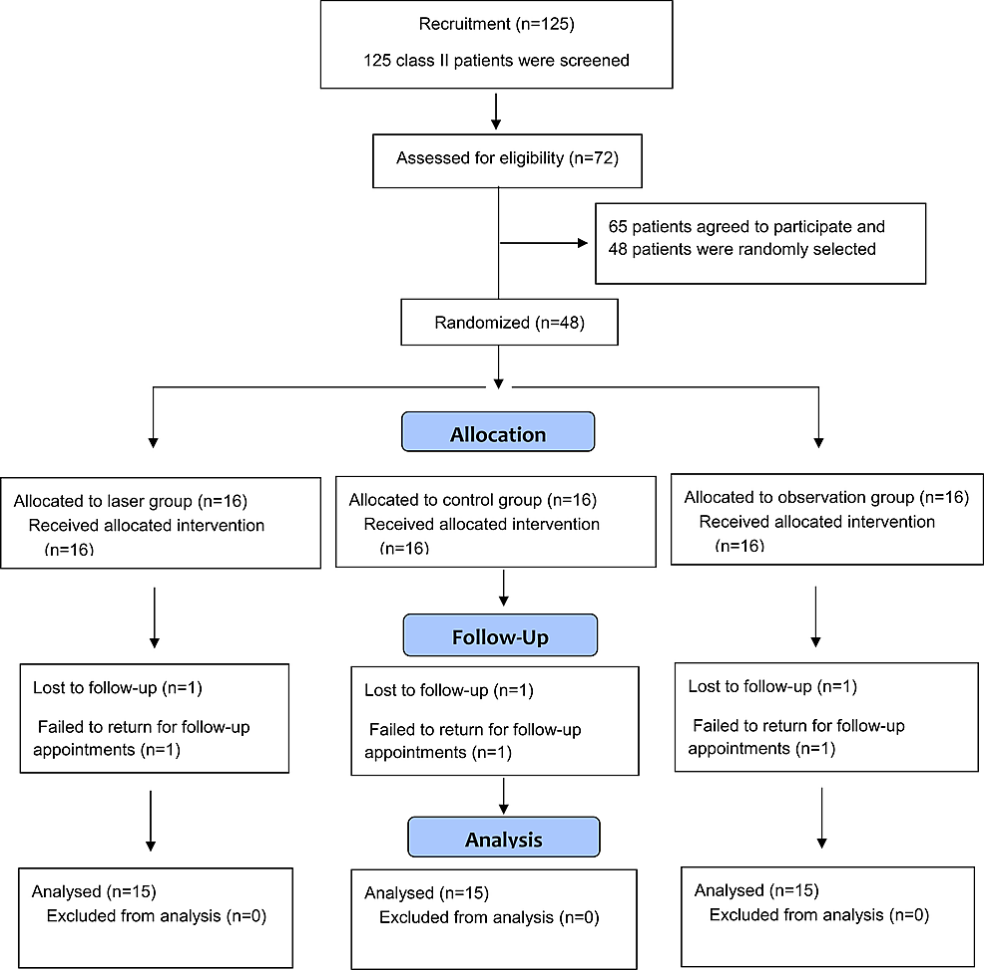
Consolidated Standards of Reporting Trials (CONSORT) flow diagram of patients' recruitment, entry into the trial, and their follow-up

Patients were selected from the patients registered in the pending records at the Department of Orthodontics, Damascus University. One hundred twenty-five patients with a primary diagnosis of Class II Division 1 malocclusion were examined, but the number of patients who met the inclusion criteria was 72. The parents of the patients were informed about the aims and methods of the study, and their informed consent was obtained once they accepted participation in the trial. Sixty-five patients agreed to participate. According to the sample size calculation, which indicated the need for 48 patients, a simple random sampling was performed to select 48 patients from the candidate patients. The inclusion criteria for the sampling frame were skeletal Class II Division 1 malocclusion resulting from mandibular retrognathia (SNB angle: 73°-78°), the sagittal skeletal discrepancy angle (ANB angle) between 4° and 9°, overjet between 5 and 9 mm, good oral health, no previous orthodontic treatment, and patients in the pubertal growth spurt peak which was assessed using hand-wrist radiograph according to the Fishman study (S or the morphological change in the epiphysis of the middle phalanx of the third finger (the middle finger) when it approached the shape of a capsule (Mp3-Cap) stage) [11]. The Consolidated Standards of Reporting Trials (CONSORT) flow diagram of patients' recruitment, entry into the trial, and their follow-up is given in Figure 1.

Randomization

The patients were divided randomly into three equal groups using a computer-generated list of random numbers with a 1:1:1 allocation ratio: (1) the Twin-Block group (TB) in which patients received Twin-Block appliances, (2) the LLLT-assisted Twin-Block group (LLLT-TB) in which patients received Twin-Block appliances supplemented with low-level laser application, and (3) the untreated control group (UCG) in which patients served as controls with no active treatment for nine months. The allocation sequence was concealed using sequentially numbered, opaque, sealed envelopes that were opened at the start of the treatment.

First group: the TB group

**Figure 2:**
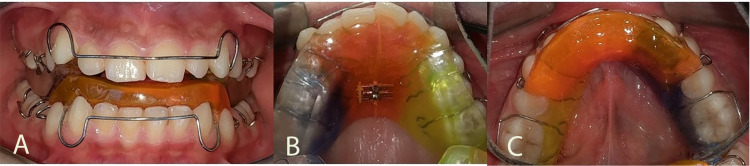
The design of the Twin-Block appliance that was used in the current study (A) A frontal view of the appliance in situ. (B) An occlusal view of the upper removable plate with an expansion screw. (C) An occlusal view of the lower plate.

Patients in this group were treated with the Twin-Block appliance and reviewed at intervals of three weeks. The functional bite construction was recorded in an edge-to-edge incisors’ relationship, which allowed an overjet up to 7 mm to be corrected with a single advancement. The mandibular opening was 2.5-4 mm which was measured clinically at the central incisors’ edges, and the slope between the upper and the lower segments of the Twin-Block was 45° (Figures 2A-2C). The patients were instructed to wear the appliances all the time except at mealtime. The upper expansion screw was turned 0.25 mm once a week until the necessary transverse expansion of the maxilla was obtained. The average amount of expansion was 12 turns (ranged from nine to 14 turns in the Twin-Block patients) which were equivalent to about 3 mm of expansion in the LLLT-TB and TB groups. The active phase of treatment was finished when a normal overjet was obtained in the retruded repeatable mandibular position (centric relation) which was assessed by the researcher [12]. After the active phase of treatment, the patients were instructed to wear the appliance for three months for retention and molar eruption, and then they continued the treatment with fixed appliances at the Department of Orthodontics, Damascus University, as needed.

Second group: the LLLT-TB group

**Figure 3:**
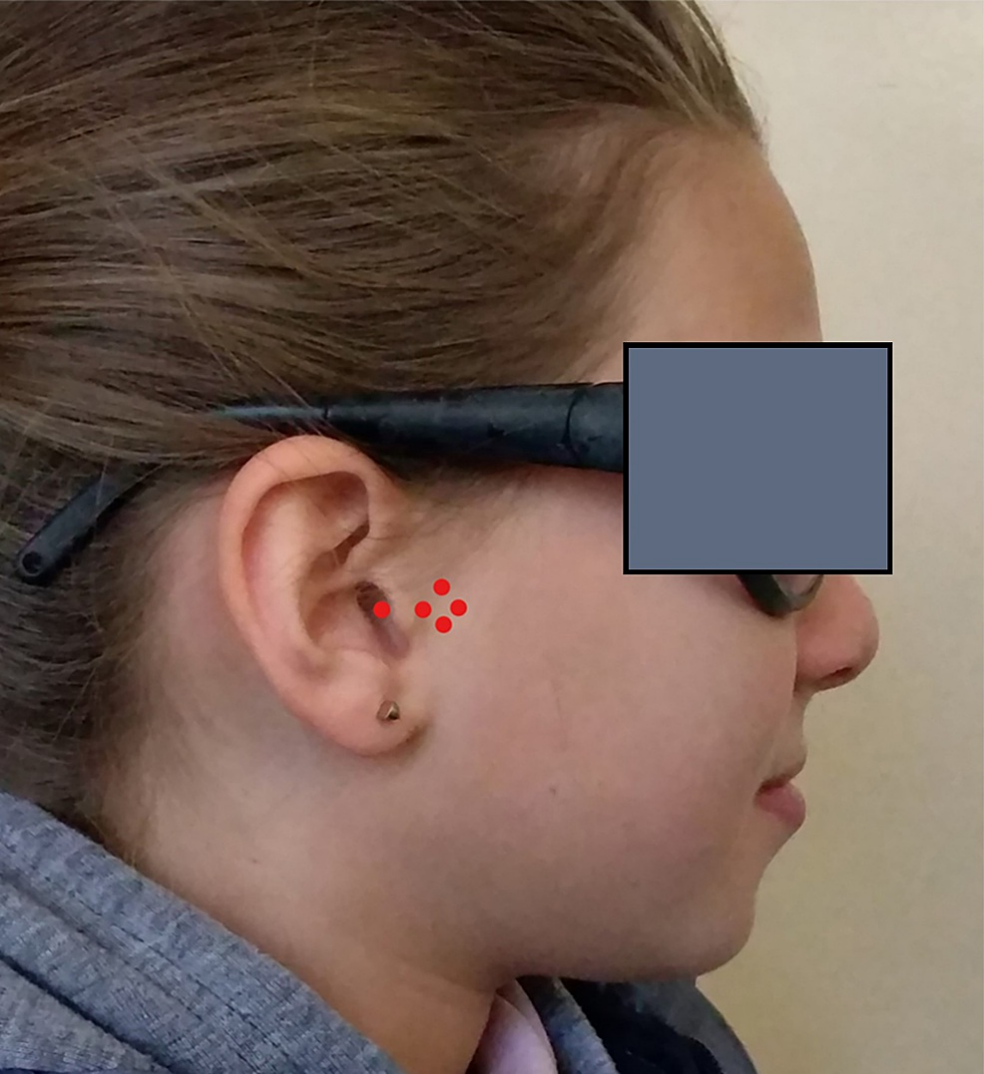
The locations of the five points irradiated by the laser beam (front, upper, lower, and behind condyle, intra-articular)

In this group, patients were treated with a Twin-Block appliance in conjunction with a low-level laser application using the low-level laser device. The treatment protocol, as well as the finishing limit of active treatment and the protocol of retention, was similar to that applied in the first group (TB group only). The low-level laser device was produced by the Konftec company (Klas-DX, Taiwan) with a wavelength of 808 nm and power of 250 mW. It was applied at the bilateral TMJ regions on the skin, at five points as north, south, east, and west (NSEW) of the condyle (front, upper, lower, and behind) and the fifth point was intra-articular (Figure 3). Each point received 5 J of the low-level laser for 20 seconds. The laser course was twice a week in the first month, every two weeks in the second month, and every three weeks until the active treatment finished. All the patients were treated by one researcher (AA).

Third Group: the UCG group

Patients in this group were observed for nine months without being subjected to any intervention. Then, the postobservation records were obtained, and they were treated at the Department of Orthodontics, Damascus University, by different MSc students under the direct supervision of one of the co-authors (ASB).

Outcome measures

Treatment Time

The treatment duration in the TB and LLLT-TB groups was calculated (in days) and was compared.

Cephalometric Variables

**Figure 4:**
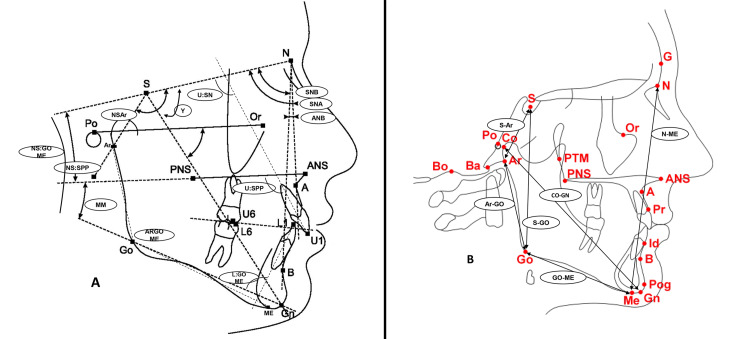
Cephalometric measurements on the tracings of the radiographs (A) Angular measurements. (B) Linear measurements. Po: uppermost point on the margin of the external auditory meatus; Y: Y-axis angle: anterior inferior angle formed by the intersection of the NS plane and the NSGn plane; A: point of most concavity on the contour of the anterior upper jaw close the root apices of the upper incisors; B: point of most concavity on the contour of the anterior lower jaw close to the root apices of the lower incisors; Or: most inferior point on the lower margin of the orbit; Bo: point at the intersection of the occipital condyle and the foramen magnum at the highest notch posterior to the occipital condyle; Ba: most anterior point on the foramen magnum; Pog: most prominent point on the anterior margin of the chin; Id: point that is located between the lower central incisors at the level of the interdental crest of the alveolus; Pr: point that is located between the upper central incisors at the level of the interdental crest of the alveolus; PTM: point located at the 11-o'clock position on the posterior border the pterygomaxillary fissure; NS: plane that connects points N and S. Abbreviations: Appendix.

Digital lateral cephalometric radiographs were taken before and after the active phase of the treatment for both interventional groups and before and after the observation period for the UCG group. Images were taken in the centric occlusion position using the same apparatus, Pax-i3D™ (Vatech Co., Hwaseong, Gyeonggi, Korea) with the same head orientation. All cephalometric radiographs were evaluated using a customized digital regimen and analysis provided by cephalometric software (Viewbox®, version 4, dHAL Software, Kifissia, Greece). The customized cephalometric analysis consisted of 14 angular variables and seven linear variables. The definitions of these measurements are given in Table 1. The definitions were derived from Jacobson [13] and Riolo et al. [14], and the performed measurements are illustrated in Figures 4A, 4B. All the cephalometric radiographs were standardized to a magnification factor which was calculated as 13%.

**Table 1:** Definitions of the angular and linear measurements used in this study Variable definitions are taken from Jacobson [13] and Riolo et al. [14]. Abbreviations: Appendix.

Variable	Definition
Angular measurements	
SNA	The angle between the anterior cranial base and the NA plane
SNB	The angle between the anterior cranial base and the NB plane
ANB	SNA minus SNB (skeletal relationship in the midsagittal plane)
SN.SPP	The angle between the anterior cranial base and the maxillary plane
SN.GoMe	The angle between the anterior cranial base and the mandibular plane
NSAr	The angle between N, S, and Ar points (the interior angle)
SArGo	The angle between S, Ar, and Go points (the interior angle)
ArGoMe	The angle between Ar, Go, and Me points (the interior angle)
Björk's sum	The sum of NSAr, SArGo, and ArGoMe angles
Y-axis	The angle between the anterior cranial base and Y-axis
MM	The angle between the maxillary plane (SPP plane) and the mandibular plane (GoMe plane)
U1.SN	The angle between the anterior cranial base and the upper incisor axis
U1.SPP	The angle between the maxillary plane (SPP plane) and the upper incisor axis
L1.GoMe	The angle between the mandibular plane and the lower incisor axis
Linear measurements	
S-Ar	The distance between S and Ar points represents the posterior cranial base
Co-Go	The distance between the Co point (point located at the most posterior and most superior contour on the condyle) and the Go point represents the ramus height
Go-Me	The distance between the Go point and the Me point represents the mandibular body length
Co-Gn	The distance between the Co point and the Gn point represents the effective mandibular length
Facial height (Jarabak's ratio)	The ratio between the posterior facial height (measured from S to Go) and the anterior facial height (measured from N to Me)
Overjet (OJ)	The horizontal overlap between the upper and lower incisors
Overbite (OB)	The vertical overlap between the upper and lower incisors

Blinding

The blinding of the researcher and the patients was not possible. However, blinding of outcome assessment was performed by an external assessor who was unaware to which group the patient belonged.

The error of the method and reliability assessment

Ten randomly selected cephalometric radiographs were remeasured after one month from the first measurement. The method error was evaluated using the intraclass correlation coefficients (ICCs) for the detection of random error and paired t-tests for the detection of systematic error.

Statistical analysis

All statistical analyses were performed using SPSS version 20 (IBM SPSS Inc., Chicago, Illinois, USA). The data were normally distributed according to Shapiro-Wilk normality tests. Gender differences among the three groups were evaluated using the chi-square test. The differences in treatment duration between the first and second groups were evaluated using an independent-samples t-test. The pretreatment variables and the changes in the three groups were compared using one-way ANOVA followed by post hoc tests for pairwise comparisons using Bonferroni's method. The changes in each group were detected using a paired-sample t-test.

## Results

Participants’ flow, entry into the trial, withdrawal, and entry to data-analysis

Forty-eight patients (20 males and 28 females) aged between 10.5 and 14.5 years were randomly assigned to the three groups. Among them, three patients (one from each group) were lost to follow-up (as shown previously in Figure 1). A total of 45 patients (15 for each group) were included in the statistical analysis.

Method error and reliability of the measurements

The results of the intraclass correlation coefficients were in the range of 0.96-0.99, which showed high reliability for the evaluated variables. No systematic errors were detected for any of the variables, i.e., no statistically significant differences were found between two sets of measurements for all evaluated variables.

Basic sample characteristics

No significant difference in the gender distribution between the two groups was observed (Table 2). At T0, there were no statistically significant differences among the groups in any variable, except for the Y-axis angle, which was slightly larger in the control group than in the LLLT-TB and TB groups (63.52°, 59.74°, and 61.19° respectively; Table 3).

**Table 2:** Gender distribution and age of the recruited patients TB: Twin-Block; LLLT-TB: low-level laser therapy-assisted Twin-Block; UCG: untreated control group; SD: standard deviation.

Sex and age		TB group	LLLT-TB group	UCG	Total	Chi-square	P-value
Males	Number	6	4	7	17	2.689	0.101
	Mean age (SD)	13.06 (1.18)	13.33 (0.71)	12.55 (0.86)	12.92 (0.92)		
Females	Number	9	11	8	28		
	Mean age (SD)	12.05 (0.98)	11.95 (1.13)	12.1 (0.91)	12.03 (0.99)		
Total		15	15	15	45		

**Table 3:** Descriptive statistics of the baseline cephalometric measurements with the P-values of significance tests of differences among the three groups *Applying one-way ANOVA. †Post hoc pairwise comparisons using Bonferroni's method. TB: Twin-Block; LLLT-TB: low-level laser therapy-enhanced Twin-Block; UCG: untreated control group; SD: standard deviation; Ns: nonsignificant. Abbreviations: Appendix.

Variable	LLLT-TB, mean (SD)	TB, mean (SD)	UCG, mean (SD)	P-value*	Pairwise comparisons P-value^†^		
					LLLT-TB\TB	LLLT-TB\UCG	TB\UCG
Angular measurements							
SNA	81.14 (4.79)	82.11 (2.58)	81.62 (2.42)	0.299	Ns	Ns	Ns
SNB	74.63 (3.42)	74.77 (1.48)	74.39 (1.97)	0.919	Ns	Ns	Ns
ANB	6.80 (1.80)	7.34 (1.92)	7.23 (0.90)	0.215	Ns	Ns	Ns
NS:SPP	7.68 (3.71)	7.89 (2.08)	8.44 (1.78)	0.736	Ns	Ns	Ns
NS:GoMe	38.46 (5.27)	37.10 (3.28)	39.01 (3.88)	0.477	Ns	Ns	Ns
NSAr	126.36 (5.52)	123.85 (5.58)	126.58 (4.15)	0.305	Ns	Ns	Ns
SArGo	143.40 (4.47)	142.46 (4.83)	141.86 (3.68)	0.643	Ns	Ns	Ns
ArGoMe	126.12 (5.86)	127.07 (6.97)	128.38 (6.81)	0.661	Ns	Ns	Ns
Björk's sum	398.46 (5.27)	397.97 (4.52)	398.44 (5.16)	0.957	Ns	Ns	Ns
Y-axis	59.74 (4.32)	61.19 (2.50)	63.52 (2.17)	0.011	0.691	0.008	0.170
MM	30.84 (5.70)	30.75 (2.77)	31.55 (4.09)	0.867	Ns	Ns	Ns
U1:SN	105.65 (5.71)	103.98 (4.76)	105.77 (2.27)	0.503	Ns	Ns	Ns
U1:SPP	113.27 (6.11)	110.00 (5.12)	114.26 (1.93)	0.055	Ns	Ns	Ns
L1:GoMe	99.12 (9.12)	97.70 (7.99)	97.73 (7.68)	0.876	Ns	Ns	Ns
Linear measurements							
S-Ar	36.04 (5.88)	37.17 (5.49)	36.06 (4.00)	0.806	Ns	Ns	Ns
Co-Go	62.39 (4.78)	61.86 (3.66)	61.99 (3.97)	0.935	Ns	Ns	Ns
Go-Me	80.96 (4.51)	82.24 (6.05)	79.56 (6.16)	0.459	Ns	Ns	Ns
Co-Gn	134.92 (9.30)	134.31 (7.48)	134.23 (6.17)	0.968	Ns	Ns	Ns
Facial height (Jarabak's ratio)	0.607 (0.072)	0.618 (0.062)	0.601 (0.056)	0.774	Ns	Ns	Ns
Overjet	6.8 (1.55)	7.73 (2.08)	7.86 (1.40)	0.209	Ns	Ns	Ns
Overbite	4 (1.41)	4.53 (1.81)	4.50 (1.37)	0.283	Ns	Ns	Ns

Treatment time

The mean treatment duration in the LLLT-TB group was 129 days, while in the TB group was 235 days; the difference between the two groups was statistically significant (P-value<0.001).

The skeletal angular differences among the three groups

Multiple comparisons among the three groups are shown in Tables 4, 5. The ANB correction was statistically significant in both LLLT-TB and TB groups (P-value<0.001). Conversely, the change in the ANB angle (mean change: -0.18°) in the control group was clinically insignificant. The interior angle formed by the intersection of the NS plane and the SAr plane (NSAr) angle decreased only in the TB group (mean change: -1.56°), while it increased in the LLLT-TB and control groups (mean change: 0.15° and 029°, respectively). There were no significant differences in vertical angles (Y-axis angle, angle between Ar, Go, and Me points (the interior angle) (ArGoMe) angle, Björk's sum, and angle between the maxillary plane and the mandibular plane (MM angle)) among the three groups.

**Table 4:** Comparisons of the changes that occurred in the cephalometric angular measurements among the three groups One-way ANOVA was used to detect significant differences among the three groups followed by Bonferroni's post hoc tests. TB: Twin-Block; LLLT-TB: low-level laser therapy-enhanced Twin-Block; UCG: untreated control group; SD: standard deviation; CI: confidence interval; Ns: nonsignificant. Abbreviations: Appendix.

Variables	LLLT-TB group	TB group	UCG	Pairwise comparisons					
				LLLT-TB\TB		LLLT-TB\UCG		TB\UCG	
	Mean (SD)	Mean (SD)	Mean (SD)	P-value	95% CI	P-value	95% CI	P-value	95% CI
SNA	-0.50 (0.47)	0.35 (0.22)	0.06 (0.19)	<0.001	-1.1466, -0.5541	<0.001	-0.8615, -0.2690	0.073	-0.5814, 0.0111
SNB	2.84 (0.41)	3.42 (1.05)	0.26 (0.09)	0.415	-2.3831, 1.180	<0.001	0.7772, 1.9797	<0.001	2.5591, 3.7616
ANB	-2.93 (0.56)	-3.08 (0.90)	-0.18 (0.17)	0.884	-1.3848, 1.5281	<0.001	-2.5195, -1.3763	<0.001	-3.4760, -2.3327
NS:SPP	-0.08 (0.52)	0.19 (0.44)	0.12 (0.33)	0.312	-0.6826, 0.1282	0.621	-0.6193, 0.1916	1	-0.3421, 0.4688
NS:GoMe	-0.24 (1.86)	0.58 (0.93)	0.19 (0.16)	0.229	-1.9437, 0.2803	1	-1.5542, 0.6698	1	-0.7225, 1.5015
NSAr	0.15 (1.47)	-1.56 (0.83)	0.29 (0.47)	<0.001	0.7782, 2.6472	1	-1.0784, 0.7906	<0.001	-2.7912, -0.9222
SArGo	-084 (4.29)	1.97 (0.95)	-0.19 (0.46)	0.017	-5.1646, -0.4733	1	-2.9892, 1.7020	0.089	-0.1703, 4.5210
ArGoMe	-0.14 (2.11)	0.11 (1.43)	-0.24 (.39)	1	-1.6364, 1.1126	1	-1.2737, 1.4753	1	-1.0118, 1.7372
Björk's sum	-0.24 (1.86)	-0.27 (0.95)	-0.13 (0.54)	1	-1.1288, 1.1720	1	-1.2683, 1.0325	1	-1.2900, 1.0108
Y-axis	-0.43 (1.54)	-0.42 (0.64)	0.13 (0.37)	1	-0.9222, 0.8948	0.391	-1.4856, 0.3314	0.418	-1.4718, 0.3452
MM	-0.20 (1.48)	-0.22 (0.55)	0.16 (0.37)	1	-0.8469, 0.8766	0.894	-1.2355, 0.4880	0.838	-1.2503, 0.4731
U1:SN	-3.68 (3.56)	-3.06 (1.78)	-0.22 (0.62)	1	-2.7576, 1.5215	0.001	-5.5930, -1.3140	0.007	-4.9749, -0.6959
U1:SPP	-3.90 (3.49)	-2.99 (0.92)	-0.32 (0.53)	0.789	-2.8422, 1.0330	<0.001	-5.5126, -1.6374	0.005	-4.6080, -0.7328
L1:GoMe	3.61 (2.34)	3.28 (1.24)	0.14 (0.45)	1	-1.0983, 1.7578	<0.001	2.0398, 4.8959	<0.001	1.7101, 4.5662

**Table 5:** Comparisons of the changes that occurred in the cephalometric linear measurements among the three groups One-way ANOVA was used to detect significant differences among the three groups followed by Bonferroni's post hoc tests. TB: Twin-Block; LLLT-TB: low-level laser therapy-enhanced Twin-Block; UCG: untreated control group; SD: standard deviation; CI: confidence interval. Abbreviations: Appendix.

Linear Measurements	LLLT-TB group	TB group	UCG	Multiple comparisons					
				LLLT-TB\TB		LLLT-TB\UCG		TB\UCG	
	Mean (SD)	Mean (SD)	Mean (SD)	P-value	95% CI	P-value	95% CI	P-value	95% CI
S-Ar	1.18 (1.30)	0.65 (0.35)	0.55 (0.12)	0.005	0.2167, 1.3100	0.001	0.3131, 1.4063	1	-0.4502, 0.6430
Co-Go	1.85 (0.93)	1.71 (0.95)	0.14 (0.12)	1	-0.5574, 0.8207	<0.001	1.0148, 2.3930	<0.001	0.8832, 2.2613
Go-Me	2.66 (1.38)	2.18 (0.71)	0.63 (0.18)	0.507	-0.7608, 2.8271	<0.001	0.6527, 4.2406	<0.001	0.3804, 3.2075
Co-Gn	4.41 (0.97)	3.66 (0.86)	1.07 (0.44)	0.047	0.0277, 1.4862	<0.001	2.6188, 4.0773	<0.001	1.8619, 3.3204
Facial height (Jarabak's ratio)	-0.00 (0.02)	-0.01 (0.03)	0.00 (0.02)	0.603	-0.0126, 0.0416	1	-0.0292, 0.0250	0.433	-0.0437, 0.0105
Overjet	-4.63 (1.24)	-5.66 (1.87)	-0.33 (0.29)	0.130	-0.1710, 2.2376	<0.001	-5.5043, -3.0957	<0.001	-6.5376, -4.1290
Overbite	-1.66 (1.31)	-2.93 (1.33)	0.20 (0.24)	0.011	0.2639, 2.2695	<0.001	-2.8695, -0.8639	<0.001	-4.1361, -2.1305

The dental angular differences among the three groups

Upper incisor retroclination was significant in the LLLT-TB and TB groups (mean change: -3.68° and -3.06°, respectively), while it was insignificant in the control group (mean change: -0.22°). Protrusion of the lower incisors was larger in the LLLT-TB group than in the TB group (mean change: 3.61° and 3.28°, respectively), while no significant protrusion was observed in the control group (mean change: 0.14°).

The linear skeletal differences among the three groups

The increases in the mandibular body length (Go-Me) and the effective mandibular length (Co-Gn) were the greatest in the LLLT-TB group compared with the TB group and the UCG group (mean change in the Go-Me: 2.66 mm, 2.18 mm, and 0.63 mm, respectively; mean change in the Co-Gn: 4.41 mm, 3.66 mm, and 1.07 mm, respectively). There were no significant differences in the ramus height (Co-Go) between the LLLT-TB and TB groups. The facial height changes were not significant among the three groups (P-value>0.05). No significant differences in overjet reduction between the LLLT-TB and TB groups were observed (mean change: -4.63 and -5.66 mm, respectively), while the reduction in the UCG was the smallest (mean change: -0.33 mm). The overbite in the LLLT-TB and TB groups was significantly reduced (mean change: -1.66 and -2.93 mm, respectively), while it increased in the UCG (mean change: 0.2 mm).

Changes over time for each group

Tables 3, 4 show the dentoalveolar changes that were observed in each group. In the LLLT-TB group, there was a significant reduction in the ANB angle (mean change: -2.93°) resulting from mandibular advancement. The upper incisors significantly retracted (mean change: -3.68°), and the lower incisors significantly protruded (mean change: 3.61°). On the other hand, the vertical angles did not differ significantly (P-value>0.05), whereas the NSAr angle increased insignificantly (mean change: 0.15°). All linear measurements increased significantly including the mandibular length (Co-Gn; mean change: 4.41 mm), the mandibular body length (Go-Me; mean change: 2.66 mm), the ramus height (Co-Go; mean change: 1.85 mm), and the posterior cranial base (S-Ar; mean change: 1.18 mm). No significant differences in the facial height index (Jarabak's ratio) were observed.

In the TB group, similar changes to those in the LLLT-TB group occurred. In addition, a significant decrease in the NSAr angle was observed (mean change: -1.56°). All linear measurements increased significantly including the mandibular length (Co-Gn; mean change: 3.66 mm), the mandibular body length (Go-Me; mean change: 2.18 mm), the ramus height (Co-Go; mean change: 1.71 mm), and the posterior cranial base (S-Ar; mean change: 0.65 mm). No significant differences in the facial height index (Jarabak's ratio) were observed.

In the UCG, the changes in the angular variables and in the overjet (OJ) and overbite (OB) were not clinically significant (P-value>0.05). All other linear measurements increased significantly including the mandibular length (Co-Gn; mean change: 1.07 mm), the mandibular body length (Go-Me; mean change: 0.63 mm), the ramus height (Co-Go; mean change: 0.14 mm), and the posterior cranial base (S-Ar; mean change: 0.55 mm), while the facial height index did not differ.

Harms

No serious harms were noticed during the course of the trial.

## Discussion

This trial is the first clinical study to evaluate the effects of low-level laser on accelerating orthodontic functional treatment and stimulating bone growth; however, the evaluation period was relatively short since patients were followed up till the end of the active treatment only. The patients were at the pubertal growth spurt so that the maximum therapeutic effects of the treatment are achieved [15].

Because there are no previous studies on functional treatment on humans, the protocol of laser application was adopted based on different studies on low-level laser and functional treatment on animals and low-level laser therapy for bone healing. A wavelength of 808 nm was employed in the current trial since it has been shown that any wavelength between 500 and 1,200 nm would be favorable to penetrate soft tissues and produce desirable effects [16], and output power of 250 mW, with 5 J per point, was used on bone biostimulation [3]. The laser was applied to the TMJ region where the condyle is the active center of growth of the mandible. We applied the laser beam on five points similar to those that were used in the protocol of pain treatment of the temporomandibular joints by Brugnera et al. [17]. These five points may have helped in irradiating considerable parts of the condyle.

The monitoring period in the UCG was chosen to be nine months because the mean period of the functional treatment with Twin-Block appliance ranges usually from six to 12 months [12,15]. Similarly, in this study, the mean treatment duration in the TB group was found to be about eight months, and it is close to the chosen monitoring period in the control group.

Because there are no previous studies on LLLT-assisted functional treatment on humans, the results were compared with the results of studies that used functional appliances solely. The treatment duration in the LLLT-TB group was almost 45% shorter than the duration in the TB group. Therefore, considering that both groups produced similar results in the correction of Class II malocclusion, a conclusion could be made that low-level laser application on the condyle region accelerated functional treatment without jeopardizing the usual dental and skeletal outcomes. Acceleration can be attributed to the ability of low-level laser on activating bone and chondral growth, and this result is aligned with the results of other studies on functional treatment with low-level laser on animals [6,9]. Therefore, a reduction of about 3.5 months in the treatment is a very promising result and may encourage using the low-level laser in daily functional treatment. Moreover, in a recent study about patients’ and orthodontists’ perceptions toward reducing treatment time [18], it was found that orthodontists would be interested to use a modern acceleration technique if it can reduce orthodontic treatment time by 20%-40%.

Many studies have reported that there is an anterior movement of the temporomandibular joint [19,20] following functional treatment depending on cephalometric measurements such as the significant decrease in the NSAr angle which has been documented in many papers. In the current trial, particularly in the TB group, the same trend was observed. However, in the LLLT-TB group, the temporomandibular joint moved a little bit posteriorly. This could probably be attributed to the larger amount of bone formation induced by the exposure to low-level laser in this group compared to the TB group.

No significant changes were observed in the facial height variables (NSGoMe, Björk's sum, Y, and MM) in the three groups. These insignificant changes are in agreement with many studies on Twin-Block appliances [12,20,21] that have reported no increase in the vertical dimension during treatment because of the observed counterclockwise mandibular rotation [20].

The upper incisors were significantly retruded in the LLLT-TB, TB, and UCG groups by a mean of -3.68°, -3.06°, and -0.22°, respectively, but this change was clinically insignificant in the control group. This retrusion is a consistent finding in previous studies on the effects of the Twin-Block appliance treatment [12,20,21] and may be due to the reaction of the upper appliance on the maxilla. The lower incisors significantly proclined by a mean of 3.61°, 3.28°, and 0.14° in the LLLT-TB, TB, and control groups, respectively, but the change in the control group was not clinically significant. This finding is in agreement with previous studies [21-23].

These results indicated that the treatment outcomes in the LLLT-TB and TB groups were similar in terms of dental and skeletal effects, despite the shortened treatment time (by almost 45%) in the LLLT-TB group compared with the TB group. This study is a short-term study, and the relapse had not been studied. To study the relapse and compare it between the LLLT-TB and the TB groups, we need a long-term period to observe the changes and the stability of the treatment.

Many animal studies [6,7,9] have reported that the application of low-level laser on the condyle increased and accelerated mandibular length growth. The current results of this study match these results; the mean effective mandibular length (Co-Gn) was statistically greater in the LLLT-TB group (4.41 mm) compared with the TB group (3.66 mm) and the UCG group (1.07 mm). Also, the mean S-Ar length was greater in the LLLT-TB group (1.18 mm) compared with the TB and the UCG groups (0.65 and 0.55 mm, respectively), which means that the application of low-level laser on the condyle region increases and accelerates bone growth. There were no significant differences in the mandibular body length (Go-Me) and the ramus height (Co-Go) between the LLLT-TB and TB groups; however, the changes were significant when compared with the control group.

Limitations

The current study employed cephalometric images in the assessment of treatment changes, but the use of three-dimensional (3D) imaging modalities such as cone-beam computerized tomography (CBCT) imaging would have provided a greater amount of knowledge about the actual 3D changes that occurred at the TMJs as well as the type and density of bone built during the observation period. It would be considered, however, unethical to obtain CBCT images of patients in the untreated control group. Another limitation of this study is that its short-term evaluation period (i.e., nine months) and longer observation periods are required to assess possible relapse in the ordinary functional treatment against the accelerated treatment. The protocol employed in the current trial could be further checked by changing and comparing other parameters of irradiation. The study was based on one functional appliance (i.e., the Twin-Block appliance), and further work should be conducted toward evaluating other removable functional appliances (e.g., Activator, Bionator, and Frankel II) and using fixed appliances to eliminate the patient compliance factor. Evaluation of patient-reported outcomes when applying a new acceleration technique has become an important issue as can be seen in many recent publications in this field [24,25]. This should be the focus of future research work. One of the possible sources of bias in the current study is related to the patients in the LLLT-TB group, who were more often present in the office, and thus, the practitioner had more possibility to motivate them to wear their appliances. Another source of bias could be linked with the researcher who was unblinded and who determined the endpoint of the active treatment in each interventional group; therefore, the conclusions of this study should be taken with caution.

## Conclusions

The application of low-level laser therapy on the condylar regions accelerated the functional treatment in Class II malocclusion patients by approximately 45% and increased the bone growth and mandibular length. The improvement in the SNB angle was similar in the LLLT-TB and TB groups. Irradiation of low-level laser stimulated bone growth at the condyles and did not cause anterior movement of the temporomandibular joint following functional orthopedic correction.

## Appendices

Abbreviations

SNA: angle between the anterior cranial base and the NA plane; SNB: angle between the anterior cranial base and the NB plane; ANB: sagittal skeletal discrepancy angle; SN.SPP: angle between the anterior cranial base and the maxillary plane; SN.GoMe: angle between the anterior cranial base and the mandibular plane; NSAr: angle between N, S, and Ar points (the interior angle); SArGo: angle between S, Ar, and Go points (the interior angle); ArGoMe: angle between Ar, Go, and Me points (the interior angle); MM: angle between the maxillary plane and the mandibular plane; U1.SN: angle between the anterior cranial base and the upper incisor axis; U1.SPP: angle between the maxillary plane and the upper incisor axis; L1.GoMe: angle between the mandibular plane and the lower incisor axis; Co-Gn: effective mandibular length; Go-Me: mandibular body length; Co-Go: ramus height; S-Ar: posterior cranial base; NA: plane that connects between points N and A; NB: plane that connects between points N and B; SN: plane that connects points N and S; NS: the plane that connects points N and S; SPP: spinal plane (the maxillary plane) which is the plane that connects points ANS and PNS; Co: most posterior and superior point on the condyle of the mandible; Gn: point that is most anterior and most inferior on the outer margin of the chin (symphysis); N: most anterior and superior point on the nasofrontal suture; S: central point in the sella turcica; Ar: point at the junction of the posterior border of the ramus and the inferior border of the body of the sphenoid bone; Go: point located the angle of the mandible between the ramus and the body of the mandible; Me: lowermost point on the margin of the chin (symphysis); U1: upper incisors; L1: lower incisors; ANS: most anterior point on the maxillary bone (anterior nasal spine); PNS: most posterior point on the maxillary bone (posterior nasal spine).
